# Three Cases of Ovarian Tumour Complicating Pregnancy, Labour and the Puerperium Respectively

**Published:** 1905-03

**Authors:** Ewen J. Maclean

**Affiliations:** Lecturer on Midwifery, University College of South Wales and Monmouthshire; Senior Gynæcologist, Cardiff Infirmary; Consulting Gynæcologist, Porth and Mountain Ash Cottage Hospitals


### THREE CASES OF OVARIAN TUMOUR COMPLICATING PREGNANCY, LABOUR AND THE PUERPERIUM RESPECTIVELY.

BY

EWEN J. MACLEAN, M.D., M.Ch. Edin., M.R.C.P. Lond., F.R.S. Edin.,

*Lecturer on Midwifery, University College of South Wales and Monmouthshire; Senior Gynæcologist, Cardiff Infirmary; Consulting Gynæcologist, Porth and Mountain Ash Cottage Hospitals.*

---

THE greater incidence of diseases of the ovary during the child-bearing period renders their occurrence as complications of pregnancy and labour far from surprising.

Ovarian disease does not afford an effective barrier to conception. Spiegelberg, for example, reports that on two occasions he examined soon after labour cancerous growths of both ovaries in which not a trace of healthy tissue remained; and Sir John Williams[1] has recorded a case in which pregnancy occurred fifteen months after one ovary had been removed for

[1] Williams, Cavendish Lecture, 1897; *West Lond. M. J.*, 1897, ii. 141.

cystic degeneration, and where towards the end of pregnancy the remaining ovary was found to be the seat of a large cystic tumour. I have myself removed two large ovarian dermoid cystomata three months after a full-time confinement.

Ovarian disease, however, tends to produce sterility. Simpson [1] estimates that while one in ten of all married women is sterile, the average of sterility in married women who are affected with ovarian disease is one in three or four. McKerron believes that the complication occurs something like once in every 2,500 pregnancies. One of the most recent contributions to the literature of the subject is the excellent and valuable monograph of McKerron,[2] of Aberdeen, whose description and analysis are based on a series of 1,290 cases, and to this source I am indebted for many of the facts quoted in this paper and for the lucidly-framed summaries of treatment. The character and site of the tumour are highly important, as these features affect the progress of pregnancy and labour, and also the puerperal convalescence.

The majority in McKerron's series were cystic, but a large proportion—viz. one in four—were dermoids, and this latter variety is for various reasons the more dangerous. Tumours of medium size are the most common. The distinction between tumours in the abdomen and tumours in the pelvis is an important one, as in the latter situation the risks are greater, especially during labour, when they form an obstruction to the passage of the child, and demand active treatment. As Sir John Williams stated in 1897, the whole evidence tends to show that the condition of pregnancy *per se* favours neither the origin nor the development of ovarian tumours.

With regard to the influence of ovarian tumour on pregnancy, it cannot be doubted that abortion is frequently caused by the mechanical effect of pressure and adhesions, and also from the supervention of complications, such as torsion of the pedicle, inflammation or rupture of the cyst, or peritonitis. Large tumours are more liable to interfere with the normal duration of pregnancy, and the larger the tumour the more likely is

[1] Simpson, *Diseases of Women*.

[2] McKerron, *Pregnancy with Ovarian Tumour*, 1903.

gestation to be interrupted. Further, from their situation in the pelvis, small tumours tend to cause incarceration of the uterus and abortion. From the pressure of the tumour, deviations in the axis of the uterus and varying degrees of peritoneal inflammation are frequently found, and the peritonitis, combined with the embarrassed respiration, favour the production of ascites.

The influence of the pregnancy on the ovarian tumour is decidedly unfavourable. The pressure of the enlarging uterus causes friction and peritonitis. Twisting of the pedicle has been estimated to occur in no less than one of every eight cases,[1] *i.e.* nearly thrice as often as in cases apart from pregnancy. This torsion may lead to rupture of the cyst from rapid distension, due to hemorrhage into its cavity, or from necrotic changes in its wall, or, again, pressure may induce the rupture. Suppuration of the cyst is also not infrequent in pregnancy, and hemorrhage into its substance, apart from torsion, has been reported. To sum up, in every fourth case some complication is to be anticipated in association with an ovarian tumour complicating pregnancy.

*Symptoms.*—A complete freedom from indicative symptoms is exceptional, though many instances have been recorded where, even in the case of large tumours, nothing unusual had been noticed until after labour had been completed, and even then the comparative lack of diminution in the abdominal dimensions has not been observed, or has been taken to indicate the presence of a second child.[2]

Pain from peritoneal irritation or stretching ot adhesions is not uncommon. Irritation of the bladder, constipation, or even obstruction from direct pressure or from adhesions have been recorded. Sudden, acute and persistent pain points to some dangerous complication.

But the symptoms associated with this condition may fail to attract special attention. In many cases the first thing to arouse the patient's suspicions has been the unusual shape or large size and rapid growth of the abdomen. In other cases attention has been attracted to the condition by the pain of

[1] McKerron, *Op. cit.* [2] See Case 3.

a sudden dislodgment upwards of a tumour from the pelvis into the abdomen.

Digestion and respiratory troubles from the distended abdomen are sometimes present, and the presence of ascites with pregnancy should always suggest the presence of an ovarian tumour.

*Diagnosis.*—When these two conditions co-exist the physical signs of the one may modify or conceal those of the other. The recognition of the pregnancy is, as a rule, the more difficult, and especially so in the early months, when the situation or size of the tumour makes bimanual palpation of the enlarged uterus difficult or impossible. The most reliable signs are changes in the cervix and the shape and consistence of the lower uterine segment. The mammary changes, especially in primiparidæ, may be helpful.

As pregnancy advances the physical signs increase in number and value. The increase in the uterine growth with intermittent contractions of its walls, ballotement, and the fœtal heart sounds are conclusive.

For the recognition of the ovarian tumour symptoms are of little use. It is on physical signs that reliance must be placed. The outline of two tumours may be recognizable, and a furrow felt between them. Ascites may be detected. Vaginal examination is of the utmost importance. The position of the cervix is rarely normal; it may be drawn high up or deviated in any of the pelvic diameters. The recognition of a pelvic tumour is also ensured by the vaginal examination. Rectal examination may be of much value, and examination under an anæsthetic may be necessary.

Differentially the signs and symptoms of the following conditions may require consideration:—Fibro-myoma uteri, retroverted gravid uterus, floating kidney, combined extra- and intra-uterine gestation, ascites, hydramnios, and pelvic exostosis.

*Prognosis.*—When an ovarian tumour complicates pregnancy the life of the woman is imperilled throughout the whole of the term.[1] The danger lies mainly in the tumour complications.

[1] Bland Sutton, *Lancet*, 1901, i. 383 *et seq.*

Such complications occur in one of every three cases. Small tumours are as dangerous as those of larger size. Early recognition is an important element in the prognosis. If the tumour is recognised and operated on before the onset of a complication the prognosis for the mother is little, if at all, more serious than in ovarian tumour apart from pregnancy. At the same time the prospects of the child are improved.

The *treatment* of ovarian tumour in pregnancy may be summarised as follows:--

1. The recognition of an ovarian tumour during pregnancy should be followed as soon as possible by ovariotomy.

2. Small tumours should be dealt with as radically as larger ones, as they are equally liable to complications.

3. Exceptionally a tumour found in the later months, and believed to be densely adherent, may be left until near the end of gestation. Its removal earlier would probably interrupt gestation.

4. Delay only increases the dangers of operation.

5. The existence of a complication arising from the tumour makes immediate ovariotomy imperative.

6. Equally good results for the mother are obtained at any month, and for the child the difference in the risks is inappreciable.

7. In the case of *dermoid* tumours the incision should be sufficiently large to allow of the tumour being removed entire.

8. If for various reasons consent to operation is postponed the advisability of operation should be especially urged, should the tumour prolapse into the pouch of Douglas.

9. Tapping of the cyst should be reserved solely for cases where symptoms of distress from distension arise, and where ovariotomy is refused or is impossible.

10. Artificial premature labour is contra-indicated in all circumstances where the urgent symptoms are due to the tumour; but where due to the pregnancy—as in nephritis—and likely to be relieved by its termination, the condition must be treated as if no ovarian disease co-existed.

**Case 1** was a married lady of 33, who had had no children and no miscarriages. She was sent to me in May, 1902, by Dr. Parsons, of Cardiff, with a diagnosis of ovarian tumour and possibly early pregnancy. These points I corroborated, and the patient, who was very anxious to have a living child, consented to operation the more readily as it was pointed out that the removal of the tumour afforded a better chance of her going to term than if the policy of non-interference were pursued. Some days later therefore, assisted by Dr. Parsons, I removed a large, mainly unilocular cystoma of the left ovary, disturbing the uterus, which was the size of 2½ months pregnancy, as little as possible. The cyst was non-adherent, and contained about two quarts of glairy, straw-coloured fluid. The remaining ovary was normal. An uneventful recovery ensued, and term was duly reached with no untoward symptoms. Dr. Parsons sent for me during the labour, as there had been considerable delay of the head at the brim, and the application of the long forceps had proved unavailing. With the axis-traction forceps a speedy delivery was effected, and the expression of the secundines was followed by a degree of *post-partum* hemorrhage. This was probably due to some interference with uterine retraction from the close adhesion of the uterus to the mid-level of the parietal wound. After gentle kneading of the uterus the bleeding ceased, and though the pulse remained rapid (115—120) for twenty-four hours, the patient made a good recovery, and is now in excellent health. Unfortunately the child died suddenly on the fourth day; the cause of death was difficult to determine. It was possibly due to intra-cranial hemorrhage following the application of the forceps.

The advent of *labour* in a woman who has an ovarian tumour brings with it special dangers. The tumour is exposed to greater pressure and risk of injury, and, further, its presence in many cases interferes with the course of the labour. The distinction here between pelvic and abdominal tumours is important. In the case of the abdominal tumours it may be stated that, as a rule, the small as well as the large tumours retard the progress of labour. The delay is usually in the second stage, and is probably due to the obliquity of the uterus, caused by the larger tumours, preventing the engagement of the head. Uterine force is wasted also by the obliquity of its axis. If there is great distension the expulsive action of the abdominal muscles is interfered with. Adhesions between uterus and tumour may prevent efficient uterine action.

The labour may seriously affect the tumour. Increased pressure may cause rupture or other injury, and torsion of the pedicle is especially liable to occur in the third stage. The breaking of adhesions between tumour and uterus may induce rupture of a cyst, as also may the abdominal manipulation by the medical attendant.

No special symptoms uniformly mark the presence of ovarian tumour during labour, though Aust Lawrence noted an unusual degree of pain. The diagnosis must rest on the physical evidence of a double tumour in the abdomen.

With regard to treatment of abdominal tumour detected during labour, everything must be done to facilitate labour, by dilatation if necessary, and to complete the delivery as soon as possible. There should thus be less risk of injury to the tumour, and care should be taken in this respect also in manipulations for the removal of the placenta.

The case must be closely watched until the removal of the tumour, which should be effected as soon as possible after labour.

The presence of an ovarian tumour in the pelvis during labour forms a serious obstruction. Early and active interference is called for in the interests of both mother and child. Unaided nature can succeed in these cases only at great risk. In very few has the existence of the tumour been suspected during pregnancy, and the excessive mortality attending these cases is probably attributable to the necessary treatment being frequently carried out under unfavourable circumstances and with insufficient assistance, as well as to the injuries to the tumour during labour.

Dermoid tumours hold a high percentage, and they are associated with the highest mortality. Rupture of the cyst occurs frequently, and "natural ovariotomy," as Playfair terms it, has resulted in the extrusion of the tumour through the torn recto-vaginal septum, or into the rectum itself or vagina. Rupture of the uterus may occur.

The diagnosis is, as a rule, unmistakable as to the presence of the tumour. Its nature may be more difficult to determine. A tumour has been taken for extra-uterine gestation.

The treatment may be summarised as follows:—

1. Ovariotomy is the best treatment if suitable environment and operative experience can be enlisted without delay.

2. If not, reposition by judicious pressure should be attempted.

3. If neither ovariotomy nor reposition be practicable, proceed without delay to aspiration, or preferably incision and packing with iodoform gauze. These measures should be followed as soon as possible by ovariotomy after labour.

4. If the tumour is solid or semi-solid, and not effectively reducible by incision, an attempt must be made to remove it by the vagina or per abdomen. If this fails, Cæsarean section must be performed.

5. Version is in all cases contra-indicated, as also are forceps and craniotomy unless the obstruction has been removed.

6. In performing the abdominal ovariotomy it should be remembered that room may be gained by delivering the fundus uteri through the wound, and a tumour may be raised out of the pelvis during the same operation by counter-pressure per vaginam.

7. Tumours aspirated or incised during labour should be removed as soon as possible in the puerperium.

**Case 2** was a primipara of 19 years, whom I was called to see at about 11.30 p.m. on July 3rd, 1903, by Dr. Henry Campbell, of Cardiff. Though the membranes had ruptured some hours previously, the head was not engaged, and was felt high up and to the right through the os, which admitted two fingers easily. The pouch of Douglas and the left pelvis were occupied by a mainly cystic tumour, which, however, presented one or two hard points—indeed, as Dr. Campbell reported, it felt, not unlike an extra-uterine fœtation. We arrived, however, at the more probable diagnosis of dermoid cystoma. The pains were regular and strong. Under chloroform I attempted to push up the tumour, but was unsuccessful. The surroundings and the untimely hour being wholly unfavourable for the removal of the tumour, after a thorough perchloride douche and various aseptic precautions I punctured the cyst per vaginam with a curved trocar and cannula, and drew off about 2½ pints of straw-coloured fluid. The tumour, now considerably reduced, could be well pushed up above the brim, but tended to prolapse when pressure was removed. The head then began to engage, and an hour or so later Dr. Campbell delivered with forceps a healthy, well-developed child, and the patient made an uninterrupted recovery.

At the Infirmary on October 5th I removed the dermoid tumour of the right ovary. The left ovary, being to all appearances healthy, was allowed to remain. The adhesions were insignificant, and the patient left the institution nineteen days later after a good recovery. The mother and child are well and strong, and the former is now pregnant for the second time and at about the sixth month of gestation. There is so far no evidence of disease of the remaining ovary.

The conditions existing *after labour* are peculiarly favourable to the development of complications in ovarian tumours, and a good recovery in the puerperium is the exception rather than the rule. At the same time it has often happened that the tumour has escaped detection, and in other instances nothing beyond the fact that the abdomen remained larger than after previous confinements has been observed.

Complications which have their origin in pregnancy or in labour may not reveal themselves clinically until the puerperium.

The most common complication is torsion of the pedicle. This is favoured by the lax abdominal wall and the sinking down of the emptied uterus to which the tumour may be attached by adhesions. Suppuration is prone to occur. Rupture of the cyst from dragging on adhesions, and inflammation of the tumour resulting in peritonitis have been reported.

The symptoms may be acute or slight and transient. They may resemble the symptoms of an acute puerperal infection, and the possibility of an ovarian cause for febrile attacks following labour should be kept in mind. An early examination with this point in view before peritonitis has masked the ovarian physical signs is of great importance in diagnosis.

Some acute complication of the puerperium complicated with ovarian tumour has been estimated to occur in one of every two cases. Here again dermoid tumours are the most dangerous, and small tumours are as much to be feared as the larger.

*Treatment of Ovarian Tumour in Puerperium.*

1. In all cases, complicated and uncomplicated alike, ovariotomy should be performed as early as possible.

2. Provided labour has been conducted with proper antiseptic precautions, the removal of the tumour may be safely undertaken within a few hours after delivery.

3. Immediate operation is imperative in all cases of acute complication.

4. Some authorities advise an exploratory laparotomy in all cases of dangerous puerperal peritonitis. An unsuspected ovarian tumour has on several occasions thus been found and removed with disappearance of all symptoms.

**Case 3** was a primipara of 32 years whom I saw, with Dr. Armstrong, of Treorky, in the fifth week of a febrile puerperium. After the delivery it was thought that the uterus was "long in contracting," and the possibility of a second child being in utero was momentarily considered. The abdomen instead of diminishing in girth had latterly been increasing, and there had been dysuria and tendency to retention of urine with various evidences of subacute peritonitis; anorexia, wasting and pyrexia. When I saw her first the physical signs were those of a fluid tumour loosely filling the lower two-thirds of abdomen and separable from the sub-involuted uterus. I corroborated Dr. Armstrong's diagnosis as to the existence of a tumour, and with his assistance I removed a very large multilocular cystoma of the right ovary. It was non-adherent, though the peritoneum was widely injected and some dark peritoneal fluid was removed. The left ovary was healthy. The patient had a considerable rise of temperature for some days prior to and during the first catamenial period following the operation, but apart from that the recovery was good and she has now regained her usual health.

